# Exploring the relation between media usage frequency and anxiety among Chinese residents: a latent profile analysis

**DOI:** 10.3389/fpsyt.2025.1475626

**Published:** 2025-03-11

**Authors:** Yi Guo, Zhuliu Gong, Ziyi Zhang, Baotong Ma, Ruitong Xia, Yuanwei Lu, Jingwen Liu, Hanjia Xin, Yumeng Cao, Saier Yang, Runqing Li, Yi Liu, Siyuan Fan

**Affiliations:** ^1^ School of Journalism and Communication, Chongqing University, Chongqing, China; ^2^ School of Stomatology, North Sichuan Medical College, Nanchong, Sichuan, China; ^3^ School of Management, Hainan Medical University, , Haikou, China; ^4^ School of Humanities and Foreign Languages, Qingdao University of Technology, Qingdao, China; ^5^ School of Management, North Sichuan Medical College, Nanchong, China; ^6^ School of Nursing, Shanxi Medical University, Taiyuan, China; ^7^ School of Nursing, Nanchang University, Nanchang, China; ^8^ Department of Stomatology, Jitang College of North China University of Science and Technology, Tangshan, China; ^9^ School of Geriatric Nursing and Wellness, Tianfu College of Southwestern University of Finance and Economic, Chengdu, China; ^10^ School of Philosophy and Social Development, Shandong University, Jinan, China; ^11^ College of Mechanical and Electrical Engineering, Qingdao Binhai University, Qingdao, China; ^12^ Department of Preventive Medicine, Yanjing Medical College, Capital Medical University, Beijing, China

**Keywords:** media use, anxiety, depression, health literacy, public health

## Abstract

**Objective:**

This study investigates the relationship between media usage patterns and anxiety levels, specifically examining how different media usage profiles influence anxiety across various demographic groups.

**Methodology:**

A total of 11,031 respondents from 120 cities across China were classified into three media usage profiles—Traditional Media-Dominant Users, New Media-Dominant Users, and Omni-Media Users—using Latent Profile Analysis (LPA) based on their media usage frequency. Demographic covariates were excluded during the initial profiling to ensure the analysis focused solely on media usage patterns. Multiple linear regression analyses were then conducted to examine the relationship between media usage types and anxiety levels. Finally, factors influencing anxiety across the different media usage profiles were explored separately.

**Results:**

The analysis revealed that both Traditional Media-Dominant and Omni-Media Users exhibited significantly higher levels of anxiety compared to New Media-Dominant Users. Factors such as geographic region, health literacy, income, debt, employment stability, and property ownership showed varying effects on anxiety across the profiles. Additionally, perceived stress and depression were identified as consistent, positive predictors of anxiety in all media usage groups.

**Conclusions:**

Compared to New Media-Dominant Users, both Traditional Media-Dominant and Omni-Media Users exhibited stronger associations with anxiety. These findings suggest that anxiety is influenced by multiple intersecting factors across media usage profiles, highlighting the need for tailored interventions that consider individuals’ specific media engagement patterns.

## 1 Introduction

Anxiety is a prevalent mental health condition that affects a significant portion of the global population. Epidemiological surveys estimate that approximately one-third of individuals will experience anxiety disorders at some point in their lives (1). Generalized Anxiety Disorder (GAD), characterized by persistent and uncontrollable worry, is a particularly common psychiatric condition. According to the World Health Organization (2), approximately 1 billion people worldwide were affected by mental health disorders in 2019. Additionally, the global prevalence of anxiety and depression increased by 25% due to the COVID-19 pandemic. In 2021, more than 33% of WHO member states reported ongoing disruptions to mental, neurological, and substance use (MNS) services, presenting unprecedented challenges to the mental health sector and widening the treatment gap for mental illnesses (3).

The COVID-19 pandemic has significantly exacerbated public anxiety, with research showing a notable increase in anxiety symptoms across multiple countries in 2021 (4, 5). Nearly one-third of adults globally reported experiencing anxiety disorders during this period (6). The high prevalence of anxiety disorders presents a serious threat to both individual and societal health, not only potentially triggering other chronic conditions but also significantly increasing the risk of suicide, further challenging public health and well-being (7–10).

In this context, the media plays a pivotal role in shaping public perceptions and emotional responses to anxiety. While media can serve as a valuable tool for disseminating information, particularly during a crisis, it also has the potential to exacerbate anxiety, especially when fear-based or sensationalized content is prevalent. Research indicates that the widespread dissemination of information during the pandemic, particularly through media channels, has contributed to heightened anxiety, stress, and depression, especially among individuals with existing or potential mental health concerns (11–14). This suggests that exposure to complex and often distressing information can trigger negative emotions, intensifying mental health issues in vulnerable individuals.

Conversely, some studies suggest that exposure to media information may also have mitigating effects on anxiety. For instance, certain types of media exposure have been shown to reduce mental health risks, including anxiety, through mechanisms such as fostering social connection or providing accurate, reassuring information (15–17). However, the impact of different frequencies and types of media consumption on public anxiety remains a complex and underexplored area. Some studies suggest that individuals who frequently engage with traditional media tend to experience lower levels of anxiety, while high-frequency exposure to new media does not necessarily have the same effect (18). This diversity in findings calls for further investigation into the nuanced relationship between media use and anxiety.

The existing literature emphasizes the need to reclassify individuals based on their media usage patterns to better understand the differentiated impacts on anxiety. Given the saturation of media in contemporary society, individuals have increasingly developed “identity bubbles,” shaped by their specific media consumption habits. Therefore, it is crucial to explore how varying frequencies and types of media exposure influence anxiety, particularly in the context of global crises such as the COVID-19 pandemic. This study seeks to address this gap by employing latent profile analysis (LPA) to identify different media usage patterns among the Chinese population and investigate how these patterns correlate with anxiety levels.

Furthermore, demographic variables such as gender (19, 20), educational attainment (21), age (22), marital status (23), debt status (22), and permanent residency (24) have all been found to influence individuals’ anxiety levels. Additionally, social support and health literacy are significant factors that modulate anxiety. Previous research has shown that higher health literacy is associated with lower levels of anxiety (25), while low social support is a well-established predictor of depression and anxiety (26, 27). Furthermore, studies consistently report a comorbidity between anxiety and depression, with individuals suffering from severe anxiety more likely to also experience severe depression (28).Therefore, another aim of this study is to investigate the correlation between sociodemographic variables, stress, depression, social support, health literacy, and anxiety among individuals with varying frequencies of media usage. This objective seeks to gain a understanding of the intricate interplay among these variables, offering scientific evidence for the development of public health policies and the establishment of mental health service systems, ultimately contributing to the improvement of individual well-being in the post-pandemic era.

## 2 Methods

### 2.1 Research object

The inclusion criteria for our study were as follows: (1) age between 18 and 60 years old, (2) nationality of the People’s Republic of China, (3) permanent resident of China with an annual overseas travel period of no more than 1 month, (4) voluntary participation in the study and completion of the informed consent form, (5) ability to complete the questionnaire survey independently or with the assistance of investigators, and (6) ability to comprehend the meaning of each item in the questionnaire. Respondents were excluded from the sample if they: (1) were suffering from mental illness or disoriented; (2) were participating in other similar research projects; (3) were not unwilling to cooperate. The Institutional Review Committee of Jinan University conducted the ethical review and approved the research plan (JNUKY-2021-018). All respondents gave informed consent and volunteered to participate in the survey.

### 2.2 Sampling method and sample quality control

This study employed a multi-stage sampling approach to obtain a geographically and demographically representative sample of participants. All provincial capital cities in China, including 23 provinces, 5 autonomous regions, and 4 municipalities directly under the central government (Beijing, Tianjin, Shanghai, and Chongqing), were included in the sampling frame. Additionally, 2 to 6 non-capital prefecture-level cities were randomly selected from each province and autonomous region using a random number table method. This resulted in a total of 120 cities distributed across eastern, central, and western regions of China, ensuring a diverse geographical representation.

Quota sampling was subsequently implemented to select participants from these cities, based on the demographic characteristics reported in the “Results of the Seventh National Population Census in 2021.” The quota sampling criteria ensured the sample reflected national population distributions in terms of age, gender, and urban-rural residence. Specifically, age distribution quotas included under 18 years old (8 ± 5%), 19–24 years old (12 ± 5%), 25–30 years old (12 ± 5%), 31–40 years old (16 ± 5%), 41–50 years old (18 ± 5%), 51–60 years old (18 ± 5%), 61–70 years old (10 ± 5%), and over 71 years old (6 ± 5%). Gender representation was maintained at an approximate 1:1 ratio of males to females, and urban-to-rural sampling followed a 3:2 ratio to ensure alignment with the urban-rural population structure. Investigators or investigation teams consisting of no more than 10 individuals were recruited in each city and received standardized training before conducting the survey. Individual investigators were responsible for collecting 30 to 90 questionnaires, while teams collected 100 to 200 questionnaires per city.

Data collection was conducted from July 10 to September 15, 2021, through the Wenjuanxing online platform (https://www.wjx.cn/). Questionnaires were distributed in a one-on-one, face-to-face manner using digital devices such as tablets or smartphones. Participants accessed the survey via a secure link, and informed consent was obtained prior to participation. For participants with cognitive capacity but physical limitations that hindered independent response, investigators conducted interviews and completed the questionnaires on their behalf. To ensure consistency and accuracy, investigators followed strict research design principles and statistical protocols during data collection. Respondents were registered and coded for analysis, and the research team conducted daily briefings to address procedural issues and ensure data quality. Weekly evaluations with investigators and teams further ensured that any discrepancies in the data were promptly resolved, maintaining the integrity and reliability of the dataset.

### 2.3 Research tool

The survey questionnaire comprised of socio-demographic information, including gender, age, region, education level, marital status, and media usage preference, as well as other factors such as social support and health literacy.

### 2.4 Self-developed media usage scale

To assess respondents’ media usage patterns, we employed a self-developed scale based on a comprehensive review of relevant literature and consultations with senior academic experts across different regions. The finalized scale included seven items measuring the frequency of contact with various media types: newspapers, magazines, radio, television, books (excluding textbooks), personal computers (including desktops, laptops, and tablets), and smartphones. Each item was scored on a 5-point Likert scale ranging from “never” (1) to “almost every day” (5), with a total possible score of 35. Higher scores indicate greater media usage frequency.

The reliability and validity of the scale were assessed through a pilot study. The Cronbach’s alpha coefficient was 0.70, indicating acceptable internal consistency. Kaiser-Meyer-Olkin (KMO) statistics were 0.74, and Bartlett’s test of sphericity was significant (p < 0.01), supporting the factor analysis. Exploratory factor analysis (EFA) extracted two factors explaining 61.80% of the total variance. Confirmatory factor analysis (CFA) indicated good model fit (CMIN/DF = 2.44, RMSEA < 0.05, GFI, TLI, IFI, and CFI > 0.90). Standardized factor loadings ranged from 0.57 to 0.87, with average variance extracted (AVE) values exceeding 0.52 and composite reliability (CR) exceeding 0.88, demonstrating good convergent validity and composite reliability.The scale has been further validated in the Chinese region (29–32).

### 2.5 GAD-7 anxiety scale

To assess the degree of anxiety severity experienced by each respondent, the study utilized the Generalized Anxiety Disorder-7 Scale (GAD-7 Anxiety Scale). (33)This scale comprises seven items that are scored on a 4-point Likert scale ranging from 0 (indicating “never”) to 3 (indicating “almost every day”). The total score of the scale is 27, with higher scores indicating greater levels of anxiety. The Cronbach’s α coefficient for this scale is 0.96, indicating high internal consistency among items.

### 2.6 Patient Health Questionnaire-9

The study employed the 9-item Patient Health Questionnaire (PHQ-9) to assess the severity of depression experienced by each respondent. (34) Respondents were asked to self-evaluate their condition based on their experiences over the past two weeks, and depression was assessed based on their self-reported scores. The scale consists of a total of nine items, scored on a 4-point Likert scale ranging from 0 (“never”) to 3 (“almost every day”), with a maximum possible score of 27. Higher scores on the PHQ-9 scale indicate a greater tendency towards depression. The Cronbach’s α coefficient for this scale is 0.94, indicating high internal consistency among items.

### 2.7 Self-developed stress scale

A self-developed stress scale was designed to measure participants’ perceived stress levels. This scale was developed following a thorough literature review and multiple expert consultations held on June 7, 11, 15, 18, and July 3 and 8, 2021. Experts holding senior academic positions from diverse regions were consulted to ensure the scale’s applicability.

The scale comprised three items: the ability to cope with stress, perceived stress over the past two weeks (including family and work stress), and perceived stress over the past year (including family and work stress). Items were scored on a 6-point Likert scale. For the first item, scores ranged from “I can overcome stress” (1) to “I am often overwhelmed by stress” (6). For the second and third items, scores ranged from “no stress” (1) to “extreme stress” (6). Total scores were calculated by summing the responses to all items, with higher scores indicating higher perceived stress. The Cronbach’s alpha coefficient for the stress scale was 0.86, indicating good reliability. The validity was supported by a KMO statistic of 0.72, and Bartlett’s test of sphericity was significant (p < 0.01), affirming the suitability of the scale for factor analysis. The scale has been further validated in the Chinese region. (35)

*Short-Form Health Literacy Instrument Scale*

The Short-Form Health Literacy Instrument Scale (HLS-SF12) was utilized to evaluate the health literacy (HL) level of the respondents. (36) This scale aimed to measure the respondents’ ability to search, comprehend, assess, and apply health-related information. The perceived difficulty of each item was rated using a Likert scale ranging from 1 to 4, where 1 meant “very difficult” and 4 meant “very easy”. The total score for all items ranged from 0 to 72. Higher scores indicated better health literacy among respondents. The HLS-SF12 had a high level of internal consistency, with a Cronbach’s α value of 0.87.

### 2.8 Statistical method

To describe continuous variables, we used mean ± standard deviation, and for categorical variables, frequencies were provided. Group comparisons were performed using the Chi-square test. Latent Profile Analysis (LPA) was conducted using Mplus 8.3 software to classify the population into distinct media use profiles based on responses to the seven-item media usage scale. During the profile identification process, demographic covariates (e.g., age, gender, income) were not included. This decision was made to allow the LPA to focus solely on identifying distinct media use patterns, uninfluenced by external variables. Following the identification of latent profiles, demographic covariates were incorporated into subsequent regression analyses to examine their association with anxiety levels and to identify potential predictors of profile membership.

The fit of the LPA was evaluated using Akaike Information Criteria (AIC), Bayesian Information Criteria (BIC), and adjusted Bayesian Information Criteria (aBIC). Lower values of these indices indicated better model fit. Additionally, entropy values, ranging from 0 to 1, were used to assess classification accuracy, with values closer to 1 indicating higher accuracy. Model fit was further evaluated using the Lo-Mendell-Rubin test (LMRT) and Bootstrapped Likelihood Ratio Test (BLRT), with p-values <0.05 indicating that the K-class model was superior to the K-1 class model. We gradually increased the number of classes until we identified the model with the best fit based on both statistical indicators and practical interpretability.

To conduct cardinality tests and perform binary logistic regression analyses of demographic factors with media usage profiles and other scales, we used SPSS 26.0 software. Statistical significance was set at a p-value of <0.05 (two-sided).

## 3 Results

### 3.1 The LPA of participants’ response to media use frequency

To determine the optimal latent profile model, we evaluated one- to six-class models using several fit indices, as presented in **Table 1**. The model fit was assessed using the Akaike Information Criterion (AIC), Bayesian Information Criterion (BIC), and adjusted BIC (aBIC). While the values of these indices initially decreased with the addition of more classes, they began to increase at the five-class model, indicating a poorer fit. Additionally, the entropy values for the three-class and four-class models were closer to 1, suggesting these models provided better classification. Both the Lo-Mendell-Rubin test (LMRT) and Bootstrapped Likelihood Ratio Test (BLRT) reached significant levels for these models, further supporting their suitability.

**Table 1:** Potential profile model fit metrics for media use frequency.

Model	K	AIC	BIC	ABIC	Entropy	LMR	BLRT	Class Probability (%)
1	14	246944.918	247047.237	247002.746				1
2	22	230380.614	230541.400	230471.487	0.919	<0.001	<0.001	0.747/0.253
3	30	221958.644	222177.898	222082.562	0.948	<0.001	<0.001	0.097/0.672/0.231
4	38	216424.795	216702.517	216581.758	0.959	<0.001	<0.001	0.089/0.115/0.668/0.128
5	46	208110.241	208446.430	208300.248	0.943	<0.001	<0.001	0.298/0.207/0.262/0.134/0.098
6	54	207582.155	207976.812	207805.207	0.985	0.994	1.0000	0.449/0.080/0.080/0.239/0.055/0.098

AIC, Akaike Information Criterion; BIC, Bayesian Information Criterion; aBIC, adjusted BIC; pLMR, p-value for LoMendell-Rubin adjusted likelihood ratio test for K vs. K-1 profiles; pBLRT, p-value for Bootstrapped Likelihood Ratio Test.

After comprehensively evaluating these indicators, we selected the three-class model as the most appropriate solution for classifying respondents’ media usage patterns. This decision was based on a balance between statistical fit and interpretability. Although the four-class and five-class models showed slight improvements in fit indices, they did not yield distinct or meaningful patterns of media usage, reducing their practical relevance. In contrast, the three-class model provided clear and interpretable profiles that aligned with theoretical expectations.

**Figure 1:**
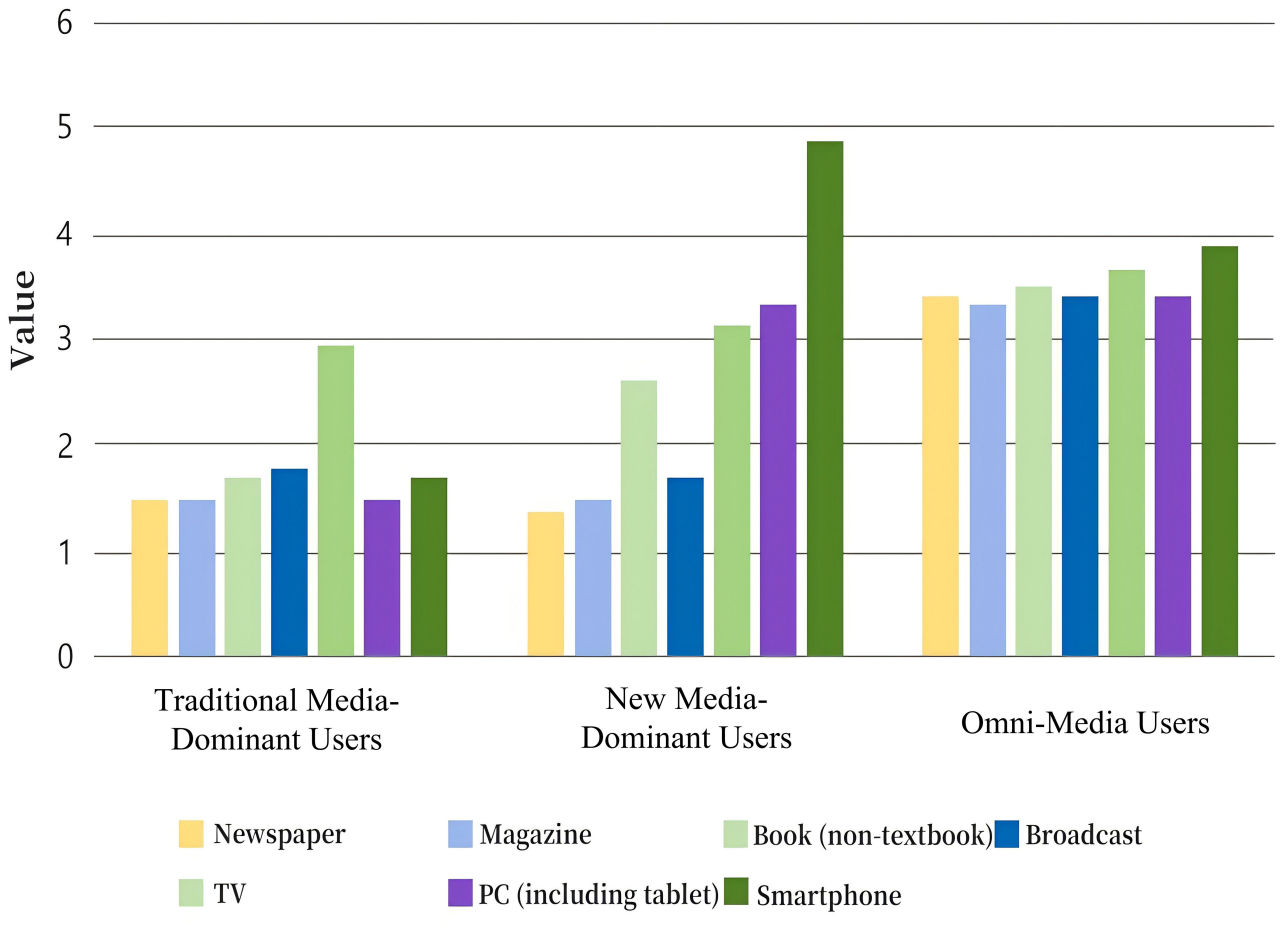
Profile of potential categories of media use frequency.

As illustrated in **Figure 1**, the three classes demonstrated distinct media usage patterns. Class 1, which we named Traditional Media-Dominant Users, accounted for approximately 9.7% of respondents and exhibited the lowest overall media usage frequency, with an average score of 12.515 ± 1.788 across the seven items. These individuals primarily relied on traditional media sources, such as television, radio, and newspapers, with minimal engagement in digital or social media. Class 2, representing 67.1% of respondents, was labeled New Media-Dominant Users due to their predominant use of new media platforms, including social media, digital news, and video streaming services, with a moderate overall media usage frequency of 18.504 ± 2.643. Class 3, comprising 23.2% of respondents, was identified as Omni-Media Users for their high-frequency engagement across all media platforms, including both traditional and new media, with an average score of 24.571 ± 3.510.

These profiles provide a nuanced understanding of distinct media consumption behaviors and offer valuable insights into how media usage patterns may relate to psychological outcomes.

### 3.2 Univariate analysis of variance for media Useg frequency

A total of 11,031 questionnaires were gathered from 23 provinces, 5 autonomous regions, and 4 municipalities throughout China. Of these respondents, 5,998 (54.4%) were women, 5,332 (42.3%) were aged between 19 and 40, 8,008 (72.6%) were urban residents, 6,360 (57.7%) were from non-agricultural households, 6,226 (56.4%) were married, and 6,487 (58.8%) were college graduates or higher education attainment. It is noteworthy that the majority of families (68.0%) had an average monthly income of less than 6,000 CNY (equivalent to approximately 833 USD). Please refer to **Table 2** for further details.

**Table 2:** Descriptive statistics and one-way ANOVA (analysis of variance).

Categories	All (N=11031, 100%)	Traditional Media-Dominant Users (n=1067,9.7%)	New Media-Dominant Users (n=7415,67.2%)	Omni-Media Users (n=2549,23.1%)	x²	p
Gender					52.017	<0.001
Female	5033 (45.6)	529 (49.6)	3147 (42.4)	1357 (53.2)		
Male	5998 (54.4)	538 (50.4)	4268 (57.6)	1192 (46.8)		
Age					166.288	<0.001
≤18	1065 (9.7)	109 (10.2)	772 (10.4)	184 (7.2)		
19-40	5332 (48.3)	257 (24.1)	3829 (51.6)	1246 (48.9)		
41-65	3759 (34.1)	318 (29.8)	2570 (34.7)	871 (34.2)		
≥66	875 (7.9)	383 (35.9)	244 (3.3)	248 (9.7)		
Region						
East	5702 (51.7)	497 (46.6)	3867 (52.2)	1338 (52.5)	76.257	0.001
Central	2987 (27.1)	298 (27.9)	1990 (26.8)	699 (27.4)		
West	2340 (21.2)	272 (25.5)	1557 (21.0)	511 (20.1)		
Nationality					41.456	0.005
the Han nationality	10386 (94.2)	1001 (93.8)	7003 (94.4)	2382 (93.5)		
Ethnic Minorities	645 (5.8)	66 (6.2)	412 (5.6)	1679 (6.5)		
Registered place of residence					35.880	0.023
Agricultural	4671 (42.3)	625 (58.6)	3018 (40.7)	1028 (40.3)		
Non-Agricultural	6360 (57.7)	4429 (41.4)	4397 (59.3)	1521 (59.7)		
permanent residence					31.8	0.062
Town	8008 (72.6)	571 (53.5)	5558 (75.0)	1879 (73.7)		
County	3023 (27.4)	496 (46.5)	1857 (25.0)	670 (26.3)		
education level					105.369	<0.001
Elementary School or Below	1127 (10.2)	453 (42.5)	481 (6.5)	193 (7.5)		
Technical secondary school/Junior high school	3417 (31.0)	340 (31.9)	2334 (31.5)	743 (29.2)		
Junior college and above	6487 (58.8)	274 (25.7)	4600 (62.0)	1613 (63.3)		
Marital status					221.0	<0.001
unmarried	4363 (39.6)	263 (24.7)	3115 (42.1)	985 (38.7)		
married	6226 (56.4)	658 (61.7)	4089 (55.1)	1479 (58.0)		
divorced	207 (1.9)	14 (1.3)	142 (1.9)	51 (2.0)		
widowed	235 (2.1)	132 (12.4)	69 (0.9)	34 (1.3)		
Religious Belief					33.1	0.045
Yes	322 (2.9)	48 (4.5)	201 (2.7)	73 (2.9)		
No	10709 (97.1)	1019 (95.5)	7214 (97.3)	2476 (97.1)		
Holding a stable job					71.371	<0.001
Yes	8835 (80.1)	605 (56.7)	6121 (82.5)	2109 (82.7)		
No	2196 (19.9)	462 (43.3)	1294 (17.5)	440 (17.3)		
monthly per capita household earning					68.1	0.007
≤6000	7500 (68.0)	861 (80.7)	5061 (68.3)	1578 (61.9)		
6001-12000	2769 (25.1)	162 (15.2)	1886 (25.4)	721 (28.3)		
>12000	762 (6.9)	44 (4.1)	468 (6.3)	250 (9.8)		
Amount of real estate					230.9	<0.001
0	1083 (9.8)	151 (14.2)	618 (8.3)	314 (12.3)		
1	6598 (59.8)	713 (66.8)	4493 (60.6)	1392 (54.6)		
2	2440 (22.1)	146 (13.7)	1706 (23.0)	588 (23.1)		
≥3	910 (8.2)	57 (5.3)	598 (8.1)	255 (10.0)		
Debt (Including Car Loans, Mortgages)					73.4	<0.001
Yes	4251 (38.5)	276 (25.9)	3034 (40.9)	941 (36.9)		
No	6780 (61.5)	791 (74.1)	4381 (59.1)	1608 (63.1)		
Depression					19781.1	<0.001
No depression	5031 (45.6)	496 (46.5)	3671 (49.5)	864 (33.9)		
Mild depression	3801 (34.5)	384 (36.0)	2722 (36.7)	695 (27.3)		
Moderate depression	1148 (10.4)	116 (10.9)	672 (9.1)	360 (14.1)		
Moderate to severe depression	803 (7.3)	56 (5.2)	273 (3.7)	474 (18.6)		
Major depression	248 (2.2)	15 (1.4)	77 (1.0)	156 (6.1)		
stress					2624.1	<0.001
Mild stress	2719 (24.6)	251 (23.5)	1946 (26.2)	522 (20.5)		
Moderate stress	7653 (69.4)	704 (66.0)	5217 (70.4)	1732 (68.0)		
Major stress	659 (6.0)	112 (10.5)	252 (3.4)	295 (11.5)		

The results of the LPA indicate that individuals with a higher frequency of media use are more likely to be men (53.2%) than women (46.8%), and are more likely to be aged between 19 and 40 (48.9%) and living in eastern China (52.5%). In terms of education level, the majority of media users (42.5%) had completed primary school or below. Interestingly, the proportion of general media users was higher in cases of no depression and mild stress, while in cases of severe depression and severe stress, the proportion of high frequency media users was higher.

The research finding shows that there were statistically significant differences among respondents in terms of gender, age, registered place of residence, region, education level, marital status, amount of real estate, debt, depression and stress (P < 0.05), indicating that these factors were influencing factors of respondents’ feelings of anxiety.

### 3.3 Summary of anxiety levels among residents

Based on the aggregation of anxiety scores, approximately 44.07% of respondents exhibited anxiety symptoms, with 13.57% classified as having moderate to severe anxiety. The remaining 55.3% reported no anxiety symptoms. Three subgroups were identified based on anxiety levels, with a total of 4,861 individuals categorized as experiencing anxiety. Among those with moderate and severe anxiety, the highest number of cases were found among Omni-Media Users, followed by New Media-Dominant Users and Traditional Media-Dominant Users, respectively.

**Figure 2:**
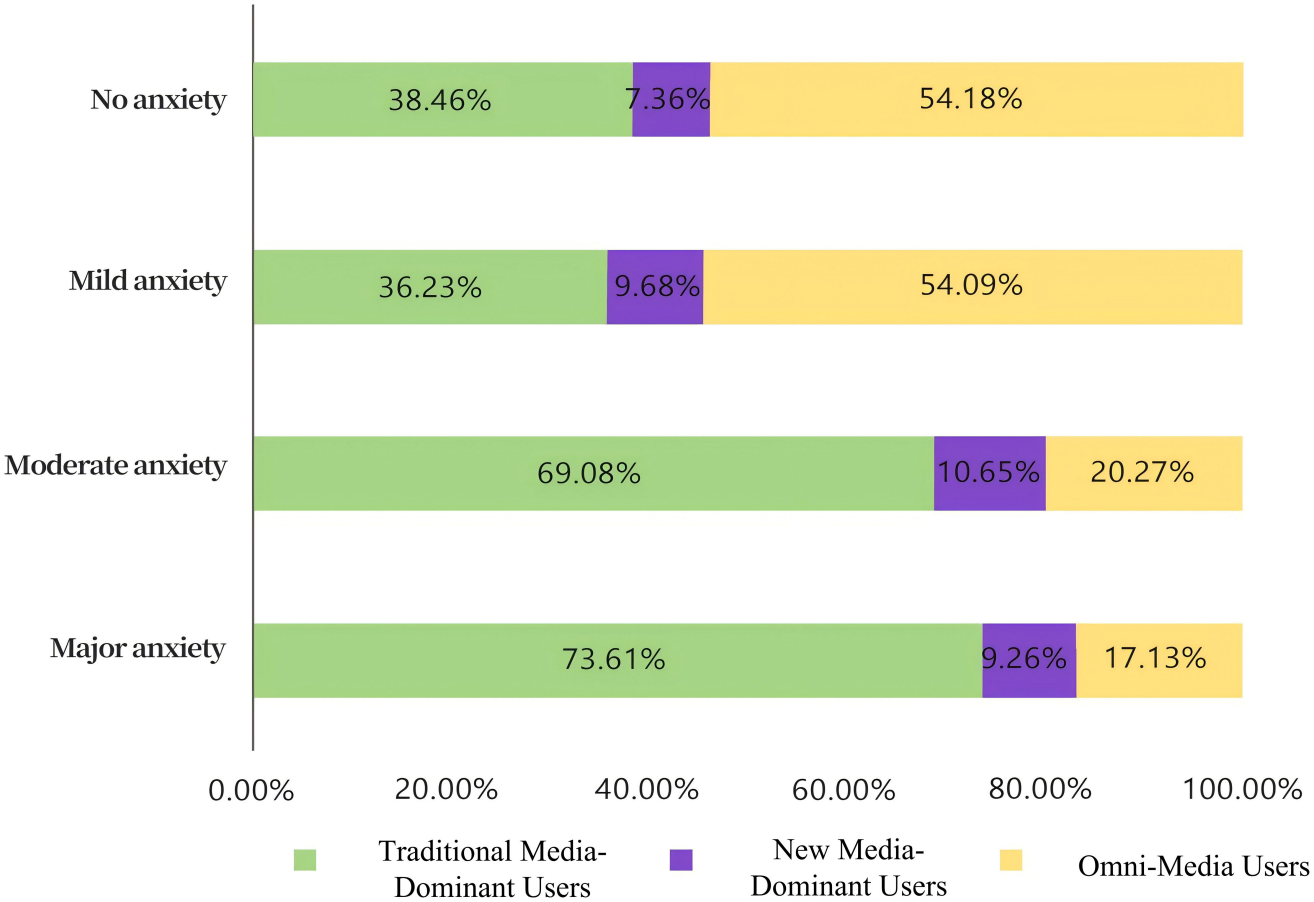
Summary chart of anxiety levels across three categories of media usage.

Specifically, 31.78% of Omni-Media Users were classified with moderate to severe anxiety, while 12.93% of Traditional Media-Dominant Users and 7.40% of New Media-Dominant Users experienced similar levels of anxiety (**Figure 2**).

### 3.4 Multivariate linear regression analysis of predictive variables on anxiety

Building on the results from the LPA, a multivariate linear regression model was used to identify predictive factors for anxiety. The dependent variable was residents’ anxiety scores, with statistical controls for variables found to have significance in the univariate analysis.

The analysis demonstrated that Omni-Media Users (*β* = 0.407, *P* < 0.001) were significantly more likely to report higher anxiety levels, suggesting a robust association between frequent media usage and increased anxiety. Other significant predictors included living in the central (*β* = 0.117, *P* < 0.05) or western regions (*β* = 0.146, *P* < 0.05), routine medication use (*β* = 0.209, *P* < 0.005), moderate to severe stress (β = 1.164, P < 0.001), and varying degrees of depression (β = 3.669 to 15.698, P < 0.001).

Furthermore, health literacy was found to negatively predict anxiety (*β* = -0.019, *P* < 0.001), with lower health literacy associated with higher anxiety levels (**Table 3**).

**Table 3:** Multivariate linear regression model of demographic social factors and other scale scores on anxiety scores.

Categories		Unstandardized Coefficients		Standardized Coefficients	*t*	*p*
		B	SE	Beta		
Independent Variable	Media(Ref:New Media-Dominant Users)					
	Traditional Media-Dominant Users	0.097	0.098	0.006	0.997	0.319
	Omni-Media Users	0.407	0.063	0.037	6.433	<0.001
Control variable	Region(Ref: East)					
	Central	0.117	0.059	0.011	1.990	0.047
	West	0.146	0.065	0.013	2.251	0.024
	Routine medication usag(Ref: No)					
	Yes	0.209	0.071	0.017	2.928	0.003
	Stress(Ref: Mild stress)					
	Moderate stress	1.164	0.062	0.115	18.842	<0.001
	Major stress	1.820	0.119	0.093	15.271	<0.001
	Depression(Ref:No depression)					
	Mild depression	3.669	0.058	0.374	62.718	<0.001
	Moderate depression	6.540	0.089	0.429	73.165	<0.001
	Moderate to severe depression	10.070	0.105	0.562	95.544	<0.001
	Major depression	15.698	0.176	0.500	89.081	<0.001
	Health literacy	-0.019	0.005	-0.025	-3.849	<0.001

Among New Media-Dominant Users, increased anxiety was linked to factors such as household monthly income between 6,000 and 12,000 CNY (*β* = 0.188, *P* < 0.005), moderate or severe stress, debt (*β* = 0.139, *P* < 0.05), and varying levels of depression. Health literacy also emerged as a protective factor, with lower levels predicting increased anxiety (*β* = -0.002, *P* < 0.001) (**Table 4**).

**Table 4:** Multivariate linear regression model of new media-dominant users on anxiety scores.

Categories	Unstandardized Coefficients		Standardized Coefficients	*t*	*p*
	*B*	*SE*	*Beta*		
monthly per capita household earning(Ref:≤6000)					
6001-12000	0.188	0.071	0.021	2.660	0.008
>12000	-0.017	0.127	-0.001	-0.132	0.895
Siblings(Ref:No)					
Yes	-0.198	0.076	-0.021	-2.627	0.009
Debt (Including Car Loans, Mortgages)(Ref: No)					
Yes	0.139	0.060	0.017	2.314	0.021
Stress(Ref: Mild stress)					
Moderate stress	1.311	0.070	0.151	18.689	<0.001
Major stress	2.540	0.172	0.116	14.789	<0.001
Depression(Ref:No depression)					
Mild depression	3.422	0.066	0.415	51.504	<0.001
Moderate depression	5.891	0.109	0.426	53.986	<0.001
Moderate to severe depression	9.403	0.160	0.446	58.825	<0.001
Major depression	14.651	0.290	0.374	50.516	<0.001
Health literacy	-0.022	0.006	-0.030	-3.628	<0.001

Conversely, for Traditional Media-Dominant Users, stress and depression were significant positive predictors of anxiety. Additionally, having a stable job (*β* = -0.458, *P* < 0.05) and owning two residential properties (*β* = -0.628, *P* < 0.05) were associated with lower anxiety levels (**Table 5**).

**Table 5:** Multivariate linear regression model of traditional media-dominant users on anxiety scores.

Categories	Unstandardized Coefficients		Standardized Coefficients	*t*	*p*
	*B*	*SE*	*Beta*		
Holding a stable job(Ref:No)					
Yes	-0.458	0.193	-0.051	-2.371	0.018
Amount of real estate(Ref:0)					
1	-0.304	0.241	-0.032	-1.261	0.208
2	-0.628	0.315	-0.049	-1.995	0.046
≥3	-0.204	0.447	-0.010	-0.458	0.647
Stress(Ref: Mild stress)					
Moderate stress	1.173	0.210	0.125	5.576	<0.001
Major stress	1.626	0.333	0.113	4.889	<0.001
Depression(Ref:No depression)					
Mild depression	4.239	0.184	0.460	22.976	<0.001
Moderate depression	7.294	0.278	0.513	26.279	<0.001
Moderate to severe depression	10.330	0.377	0.520	27.378	<0.001
Major depression	13.764	0.693	0.366	19.864	<0.001

For Omni-Media Users, moderate to severe stress and depression were key predictors of anxiety. However, health literacy (*β* = -0.034, *P* < 0.005) remained a significant negative predictor for anxiety in this group (**Table 6**).

**Table 6:** Multivariate linear regression model of omni-media users on anxiety scores.

Categories	Unstandardized Coefficients		Standardized Coefficients	*t*	*p*
	*B*	*SE*	*Beta*		
Region(Ref: East)					
Central	0.261	0.131	0.020	1.995	0.046
West	0.290	0.149	0.020	1.946	0.052
Stress(Ref: Mild stress)					
Moderate stress	0.481	0.152	0.039	3.167	0.002
Major stress	0.819	0.219	0.045	3.747	<0.001
Depression(Ref:No depression)					
Mild depression	4.244	0.151	0.325	28.016	<0.001
Moderate depression	7.764	0.194	0.465	40.074	<0.001
Moderate to severe depression	10.926	0.174	0.731	62.630	<0.001
Major depression	16.931	0.268	0.698	63.177	<0.001
Health literacy	-0.034	0.012	-0.035	-2.878	0.004

## 4 Discussion

This study empirically analyzed the latent categories of media usage patterns and their impact on anxiety levels among the Chinese population. The findings reveal that the Chinese public’s media exposure patterns can be categorized into three distinct groups: Traditional Media-Dominant Users, New Media-Dominant Users, and Omni-Media Users. These categories significantly differ in terms of their relationship to anxiety, particularly when compared to New Media-Dominant Users.

Our findings align with existing literature, which indicates that the frequency and type of media exposure significantly impact mental health outcomes, particularly anxiety (37). Media richness theory suggests that increased exposure to multiple media types can lead to cognitive overload and negative psychological outcomes (38). This is particularly relevant during the COVID-19 pandemic, where excessive exposure to news across various media platforms has been shown to exacerbate anxiety levels (39). Similarly, Nourisaeed et al. (40) reported that excessive engagement with online health information during the pandemic contributed to heightened anxiety symptoms. Additionally, the rapid spread of misinformation, conspiracy theories, and rumors has been described as an “infodemic,” which amplified public perceptions of the COVID-19 threat and exerted significant psychological pressure on the general population (41, 42). Therefore, individuals with higher media exposure frequencies are more susceptible to anxiety, which further supports the relationship between media usage frequency and anxiety.

This study also highlighted the presence of distinct factors affecting anxiety among individuals who reported differing media usage patterns. It uncovered that individuals who reported moderate frequency of media use displayed anxiety symptoms that were influenced by several factors including family income per capita, debt status, health literacy, and social support. Social support refers to an individual’s perception of the external support they receive or can receive. This study found that the lower level of social support, the more anxiety an individual with moderate frequency of media use may experience. It also uncovered that despite the varying frequencies of media usage across different groups of people, stress and depression positively predict the anxiety levels. This finding aligns with previous research, which found that individuals with higher levels of perceived stress tend to experience symptoms of depression and anxiety. (43) Furthermore, those with a pre-existing mental health condition are more likely to suffer from psychological distress. (44) The findings suggest that vulnerable individuals may require additional social support to mitigate the risk of anxiety associated with media use.

When reconsidering media usage and anxiety, it is important to take into account the influence of health literacy as a significant factor. This study revealed that among individuals who consume mass media more frequently, health literacy significantly negatively predicts the anxiety levels. In other words, the higher health literacy an individual possesses, the less anxious they were when exposed to media. This finding is consistent with previous studies conducted in other countries. For instance, Olagoke et al. found that higher health literacy correlated with lower levels of future anxiety in Polish adult Internet users. (45) Similarly, McCaffery et al. conducted a survey of Australian adults and discovered that inadequate levels of health literacy were associated with lower perceived threat severity and higher levels of anxiety. (46)

Our study reveals that media consumption patterns—categorized into Traditional Media-Dominant Users, New Media-Dominant Users, and Omni-Media Users—highlight the inadequacy of a one-size-fits-all approach. Tailored interventions, addressing the unique media behaviors of each group, are essential for effectively managing anxiety. For Traditional Media-Dominant Users, strategies should focus on balanced media consumption, limiting exposure to sensationalized content, and enhancing media literacy. New Media-Dominant Users, more vulnerable to the psychological effects of digital media, would benefit from interventions such as digital detox, content regulation, and media literacy to distinguish credible from misleading information. Omni-Media Users, at risk of media overload, require personalized media consumption strategies and mindful practices to manage anxiety. Across all groups, improving health literacy is a key cross-cutting intervention, empowering individuals to critically engage with media and reduce its psychological impact. Public health initiatives aimed at enhancing health literacy could significantly improve mental well-being and reduce anxiety among diverse media users.

## 5 Conclusion

This study explores the relationship between media usage patterns and anxiety levels among the Chinese population, identifying three distinct media usage profiles: Traditional Media-Dominant Users, New Media-Dominant Users, and Omni-Media Users. The results indicate that, compared to New Media-Dominant Users, Omni-Media Users are a significant positive predictor of anxiety levels, suggesting that higher media exposure is associated with elevated anxiety. Additionally, sociodemographic factors such as geographic region, health literacy, income, debt, and employment stability were found to influence anxiety levels across these profiles. Perceived stress and depression emerged as consistent predictors of anxiety, underscoring their central role in mental health.

## 6 Limitation

This study has several limitations. First, while conducted in Mainland China during the 2021 COVID-19 pandemic, the findings may not be generalizable to other contexts. Although quota sampling based on national census data was employed, rural and remote populations were underrepresented, potentially affecting the generalizability of results. Second, the exclusion of adolescents (<18 years) and older adults (>60 years) limits the applicability of the findings to these age groups, which may exhibit distinct media usage patterns and anxiety responses. Third, reliance on self-reported data and a cross-sectional design precludes causal inferences and introduces potential biases such as recall error and social desirability. Fourth, unmeasured factors such as social isolation, information overload, or pre-existing anxiety may confound the observed associations. Finally, media usage was treated as a general construct without distinguishing between different media types (e.g., social media, news, entertainment), each of which may have distinct psychological impacts. This oversimplification limits the precision of the findings and their practical applicability.

To address these limitations, future studies should aim to recruit a more geographically and demographically representative sample, include diverse age groups, account for potential confounders, and differentiate between specific media types to provide more nuanced insights.

## Data Availability

The original contributions presented in the study are included in the article/supplementary material. Further inquiries can be directed to the corresponding authors.
